# The integrated analysis of PRDX3 in lung cancer: biomarker potential and therapeutic target prospects

**DOI:** 10.1186/s12931-026-03703-5

**Published:** 2026-05-06

**Authors:** Qi Kong, Lei Zhang, Shuang Gong, Yue Wu, Jue Wang

**Affiliations:** 1 Institute of Laboratory Animal Sciences, Comparative Medicine Center, Chinese Academy of Medical Sciences (CAMS), Peking Union Medical College (PUMC), Beijing, 100021 China; 2 NHC Key Laboratory of Comparative Medicine, State Key Laboratory of Respiratory Health and Multimorbidity, Key Laboratory of Pathogen Infection Prevention and Control (Peking Union Medical College), National Human Diseases Animal Model Resource Center, National Center of Technology Innovation for Animal Model, Ministry of Education, Beijing, 100021 China

**Keywords:** Lung cancer, Ferroptosis, PRDX3, Gene expression, Prognostic biomarker, Redox homeostasis, Tumor immune microenvironment

## Abstract

**Background:**

Lung cancer remains the malignant tumor with the highest global incidence and mortality, posing a severe threat to public health. Peroxiredoxin 3 (PRDX3), a well-characterized ferroptosis biomarker, has been implicated in lung cancer progression. Accumulating evidence suggests that PRDX3 may serve as a core gene and potential biomarker, which is critical for elucidating pathogenesis, improving diagnostic accuracy, and developing targeted therapeutic strategies for lung cancer.

**Methods:**

To systematically investigate the expression pattern of PRDX3 and its clinical relevance in lung cancer, we integrated multiple bioinformatics tools and public datasets to analyze PRDX3 expression across pan-cancer cohorts, major lung cancer subtypes, and patient subgroups stratified by sex, age, and clinical stage. Furthermore, we evaluated the correlation between PRDX3 expression and immunotherapeutic-related factors, and performed multivariate survival analysis to validate its independent prognostic value. Additionally, protein-protein interaction (PPI) networking and co-expression analysis were employed to explore the functional interactions of PRDX3 with core ferroptosis regulators and immune microenvironment modulators.

**Results:**

PRDX3 was significantly upregulated in multiple malignant tumors, including LUAD and LUSC, with minimal variations in expression levels across different sex, age, and clinical stage subgroups. High PRDX3 expression was independently associated with significantly poorer clinical prognosis in lung cancer, particularly in the LUAD subtype. Pathway enrichment analysis revealed that immune activation, immune inhibition, and MHC molecule-related signaling pathways were closely correlated with altered PRDX3 expression in both LUAD and LUSC, with distinct subtype-specific patterns. PPI networking demonstrated that PRDX3 exhibited positive co-expression with core ferroptosis regulators (GPX4, TXN2) and interacted with components of the thioredoxin system (TXNRD2, TXN), indicating its potential involvement in regulating cellular redox homeostasis and ferroptosis. Single-cell analysis further confirmed PRDX3 expression in lung cancer cells and key immune cell subsets (T cells, macrophages), with higher expression levels observed in LUAD than in LUSC.

**Conclusions:**

These correlative findings suggest that PRDX3 may play a pivotal role in lung cancer pathogenesis, potentially through mechanisms involving ferroptosis regulation via interaction with GPX4 and the thioredoxin system, redox homeostasis maintenance, and subtype-specific immune microenvironment modulation. Collectively, PRDX3 represents a promising independent prognostic biomarker for lung cancer, especially for LUAD, and a potential therapeutic target for ferroptosis-based and immunotherapeutic strategies.

**Graphical abstract:**

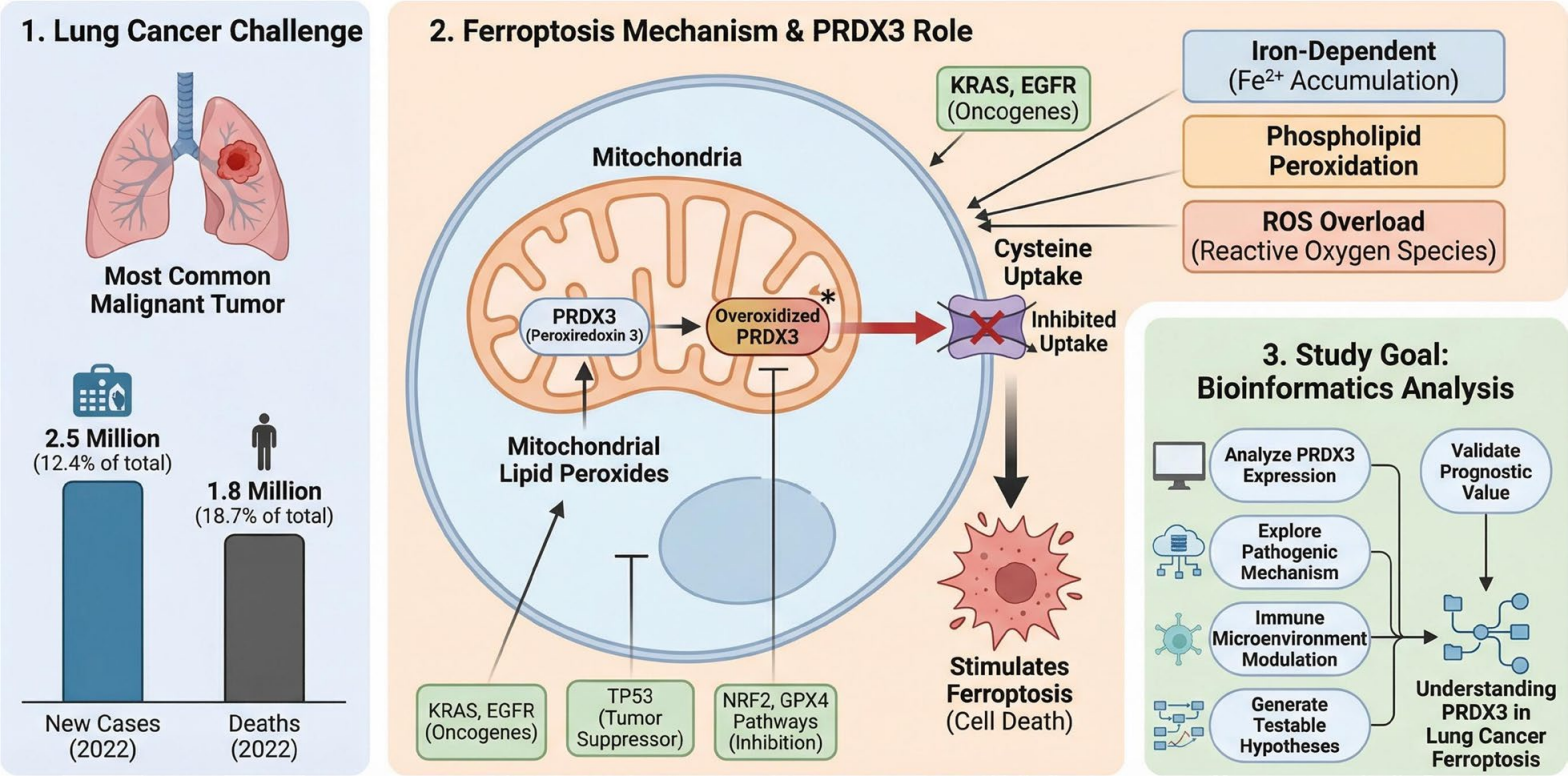

## Introduction

Lung cancer, originating from tracheal, bronchial mucosal, or glandular tissue, is the most prevalent malignant lung tumor worldwide. According to the 2022 global cancer statistics, approximately 2.5 million new lung cancer cases were diagnosed, accounting for 12.4% of all new cancer cases, and 1.8 million deaths were attributed to lung cancer, representing 18.7% of all cancer-related deaths [1].

Ferroptosis is an iron-dependent form of regulated cell death, characterized by phospholipid peroxidation, intracellular free iron accumulation, and reactive oxygen species (ROS) overload [2]. Multiple oncogenes (e.g., KRAS, EGFR), tumor suppressors (e.g., TP53), oncogenic signaling pathways, and ROS signaling cascades are involved in ferroptosis regulation, which has been identified as a critical targeted vulnerability in cancer [3]. Lung cancer is closely associated with ferroptosis: ferroptosis inducers can effectively promote lung cancer cell death [4], and targeted modulation of iron metabolism and the P53-NRF2-GPX4 axis can induce ferroptosis to inhibit lung cancer cell growth and proliferation [5]. Notably, ferroptosis is involved in all stages of lung adenocarcinoma (LUAD) initiation, proliferation, and progression, making its regulation a novel direction for lung cancer prevention and treatment [6].

Peroxiredoxin 3 (PRDX3), a mitochondrial peroxidase, is hyperoxidized by mitochondrial lipid peroxides in ferroptotic cells and can stimulate ferroptosis by inhibiting cysteine uptake. Previous studies have shown that hepatic PRDX3 expression is positively correlated with liver injury markers (AST, ALT) [7], and its role in ferroptosis across different tissues highlights its potential as a ferroptosis biomarker. Despite the well-established association between ferroptosis and lung cancer, the expression patterns, clinical relevance, and potential mechanistic roles of PRDX3 in lung cancer ferroptosis and progression remain largely unelucidated. In the present study, we performed a comprehensive bioinformatics analysis to characterize PRDX3 expression in lung cancer, validate its prognostic value, and explore its potential correlative links to ferroptosis regulation and immune microenvironment modulation, thereby generating testable hypotheses for future experimental validation.

## Materials and methods

### Analysis of PRDX3 gene expression data in pan-cancer

TIMER (https://cistrome.shinyapps.io/timer) and the Human Protein Atlas (HPA, https://www.proteinatlas.org/ENSG00000165672-PRDX3) were used to evaluate PRDX3 mRNA and protein expression levels across pan-cancer cohorts [8]. Wilcoxon rank-sum tests were employed to compare PRDX3 mRNA levels between tumor and adjacent normal tissues, with statistical significance defined as *p* < 0.05. For HPA analysis, the frequencies of PRDX3 genetic alterations across pan-cancer samples were quantified, and “deep deletion” was defined as homozygous loss of the PRDX3 gene locus in accordance with HPA database annotations.

### Analysis of PRDX3 gene expression data in lung cancer patients

UALCAN (https://ualcan.path.uab.edu/cgi-bin/CPTAC-Result.pl? genenam=PRDX3&ctype=LUAD) was used to analyze PRDX3 expression in LUAD and LUSC, including subgroup analyses stratified by sex, age, race, smoking status, and clinical stage [9]. Wilcoxon rank-sum tests were applied to compare PRDX3 expression between tumor and normal tissues, as well as between different patient subgroups.

### Clinical survival analysis of PRDX3 and lung cancer patients

Kaplan-Meier Plotter (https://www.kmplot.com/analysis/index.php? p=service) was used to generate univariate Kaplan-Meier survival curves for PRDX3 high-expression and low-expression groups in lung cancer, LUAD, and LUSC [10]. Multivariate Cox proportional hazards regression analysis was performed to assess the independent prognostic value of PRDX3 expression, with adjustments for potential confounding factors (sex, age, clinical stage, smoking status). Survival curves were plotted with time (months) as the x-axis and overall survival rate as the y-axis. Log-rank tests were used to determine the significance of univariate survival analysis, and Wald tests were employed for multivariate Cox regression analysis, with statistical significance defined as *p* < 0.05.

### Meta-analysis of PRDX3 gene in lung cancer patients

Lung Cancer Explorer (LCE, https://lce.biohpc.swmed.edu/lungcancer/metaresult.php? jobid=68ab0f584d2524.09583174&geneinfo=PRDX3), a lung cancer-specific database containing clinical and expression data from more than 6700 patients, was used for PRDX3 meta-analysis [11]. Meta-analysis of PRDX3 expression differences between tumor and non-malignant tissues, as well as the correlation between PRDX3 expression and overall survival, was performed using random-effects models. Heterogeneity across cohorts was assessed via I² statistics and *p*-values for heterogeneity.

### The impact of immune factors on PRDX3 gene expression

TISIDB (https://www.biosch.hku.hk/TISIDB/index.php, not available at present), a tumor-immune interaction database integrating high-throughput data and published literature, was used to analyze the correlation between PRDX3 expression and immune modulators (immunosuppressants, immunostimulants, MHC molecules) in LUAD and LUSC [12].Spearman rank correlation analysis was performed to quantify the correlation between PRDX3 and immune modulators, with Benjamini-Hochberg false discovery rate (FDR) correction applied for multiple testing. Statistical significance was defined as FDR-adjusted *p* < 0.01. Immune cell infiltration correlation analysis was additionally performed using TIMER to explore the association between PRDX3 expression and the infiltration levels of key immune cell subsets (CD8 + T cells, M2 macrophages, Tregs, neutrophils) in LUAD and LUSC.

### Single-cell analysis of PRDX3 expression on the integrated metacell UMAP plots

TE-SCALE (https://ngdc.cncb.ac.cn/te-scale/tissue-map/lung), a comprehensive single-cell database for analyzing gene and transposable element expression in human cancers, was used for single-cell PRDX3 expression analysis [13]. PRDX3 expression was visualized on integrated metacell UMAP plots, with annotations for cancer type (LUAD/LUSC), cell type (tumor cells, T cells, macrophages, B cells), sex, tissue site, and tumor stage. The expression levels of PRDX3 in distinct cell subsets were quantified and correlated with immune modulator expression (from Sect. 2.5) to establish a link between single-cell expression patterns and the tumor immune microenvironment.

### Functional analysis of PRDX3 protein molecules

Protein-protein interaction (PPI) networks for PRDX3 were constructed using STRING (https://cn.string-db.org/cgi/network? taskId=bg9Gtfn3n8LZ&sessionId=b7mAjgIQ3fUO) (confidence score > 0.7) and GeneMANIA (https://genemania.org/search/homo-sapiens/PRDX3) [14]. Gene Ontology (GO) and Kyoto Encyclopedia of Genes and Genomes (KEGG) enrichment analyses were performed on PRDX3-interacting proteins via STRING, with functional categories limited to biological processes (BP), molecular functions (MF), and pathways related to ferroptosis, redox homeostasis, and immune signaling.

Co-expression analysis of PRDX3 and core ferroptosis/immune genes was performed using ENCORI/starBase (https://rnasysu.com/encori/panGeneCoExp.php), with Pearson correlation analysis (|r|>0.3, *p* < 0.001) used to identify significant co-expression relationships in LUAD and LUSC. Core ferroptosis genes analyzed included *GPX4*,* ACSL4*,* SLC7A11*,* TXN2*,* TXNRD2*, and *TP53*; immune genes included *IL10*,* TGFB1*,* CXCL12*,* B2M*, and *TAP1*.

### Statistical methods

All statistical analyses were performed using R software (version 4.3.1) and the built-in statistical modules of the bioinformatics tools employed. Wilcoxon rank-sum tests were used for two-group comparisons of gene expression levels. Spearman rank correlation analysis was utilized to assess the correlation between PRDX3 and immune modulators (with FDR correction), and Pearson correlation analysis was employed for gene co-expression analysis. Univariate survival analysis was conducted using log-rank tests, and multivariate prognostic analysis was performed using Cox proportional hazards regression (adjusted for sex, age, clinical stage, and smoking status). Meta-analysis was carried out using random-effects models, with heterogeneity assessment via I² statistics. Statistical significance was defined as *p* < 0.05 (uncorrected) or FDR-adjusted *p* < 0.01 (for multiple testing). PRDX3 was considered upregulated or downregulated in tumors if the fold change was greater than 1.5 and *p* < 0.05.

## Results

### Pan-cancer analysis of PRDX3 gene expression data

**Fig. 1:**
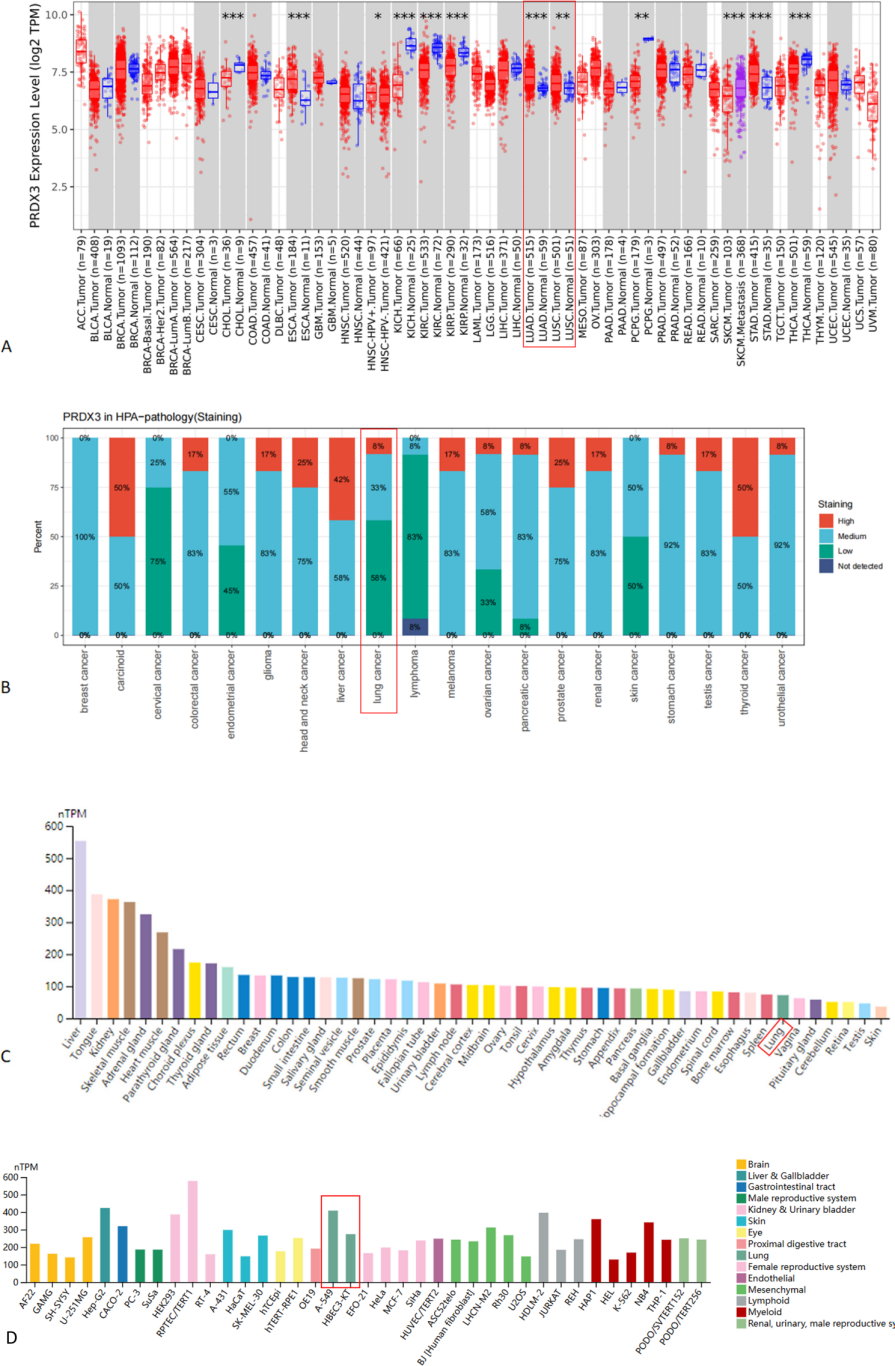
PRDX3 gene expression in pan-cancer. **A** Differential expression of PRDX3 gene in pan-cancer (Wilcoxon, *: *p* < 0.05; **: *p* < 0.01; ***: *p* < 0.001); **B** The proportion of PRDX3 genetic alterations in pan-cancer samples (HPA database; deep deletion = homozygous loss of PRDX3 locus); **C** PRDX3 RNA tissue-specific expression in normal human tissues; **D** PRDX3 RNA tissue-specific expression in lung tumor tissues (the Human Protein Atlas)

Analysis of the TIMER database revealed that PRDX3 was significantly overexpressed in four cancer types, including esophageal cancer (ESCA), LUAD, LUSC, and gastric adenocarcinoma (STAD) (Wilcoxon test, *p* < 0.001; Fig. 1A), indicating that PRDX3 may play a conserved role in the pathogenesis of these malignant tumors.

HPA database analysis of PRDX3 genetic alterations in The Cancer Genome Atlas (TCGA) pan-cancer samples showed that the highest alteration frequency was observed in lung cancer, with the dominant alteration type beingdeep deletion (homozygous loss of the PRDX3 locus, Fig. 1B). Despite the high frequency of deep deletion, PRDX3 mRNA and protein overexpression was observed in lung cancer tumor tissues (Fig. 1A and C), suggesting potential dose-dependent or compensatory upregulation of the remaining PRDX3 allele in tumor cells with deep deletion, or context-dependent regulation of PRDX3 in lung cancer. HPA RNA tissue-specific expression analysis confirmed that PRDX3 is highly expressed in the lung relative to other normal tissues and tumor tissues such as A549 cell and HBEC3-KT cell (Fig. 1C-D), which is consistent with a tissue-specific role of PRDX3 in lung homeostasis and disease progression.

### Analysis of PRDX3 gene expression data in patients with lung cancer

**Fig. 2:**
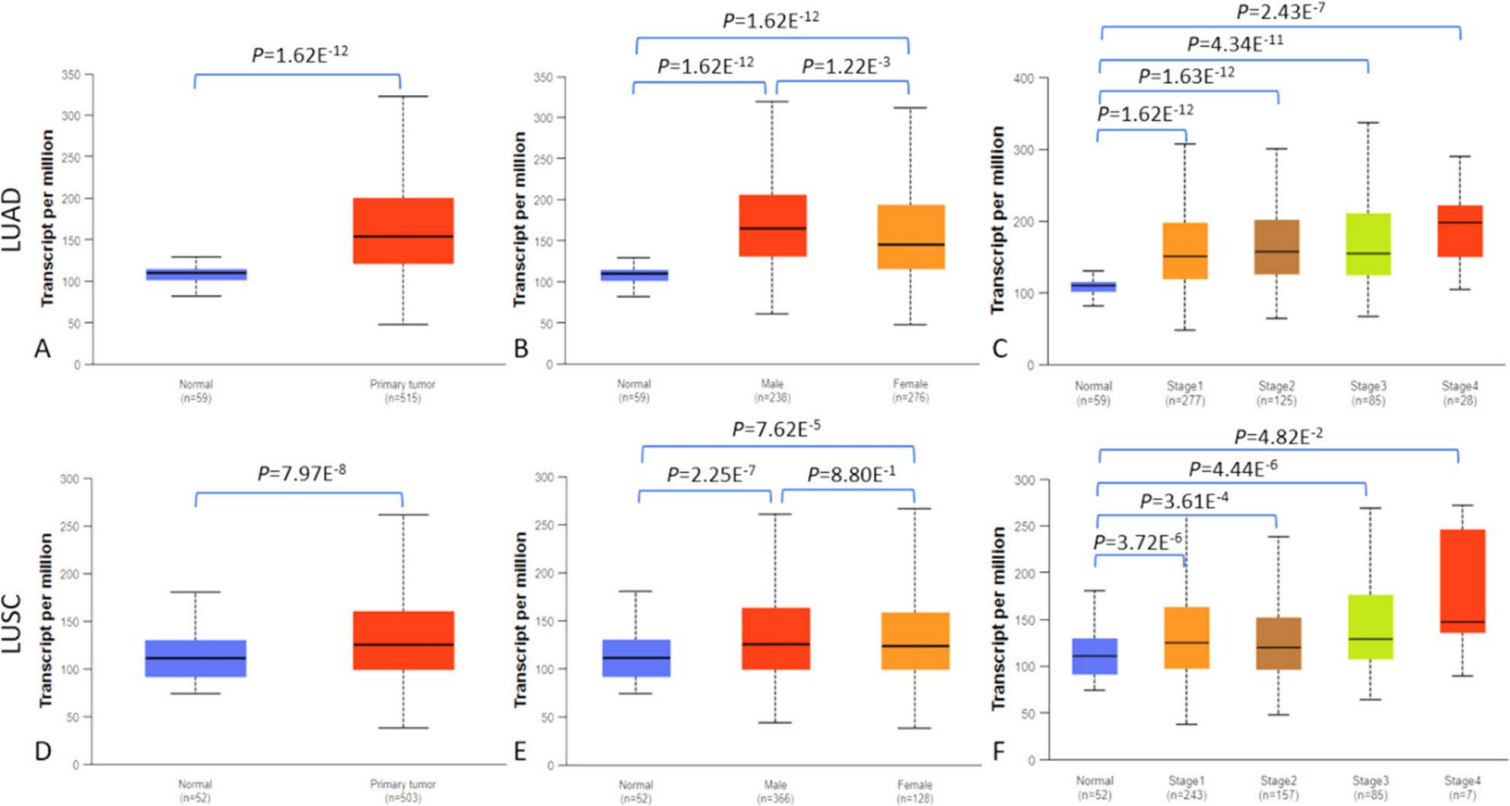
Expression of PRDX3 gene in LUAD and LUSC. **A** PRDX3 expression in LUAD tumor vs. normal tissue (*P* = 1.62E^− 12^); **B** PRDX3 expression in LUAD by sex (tumor vs. normal); **C** PRDX3 expression in LUAD by clinical stage (tumor vs. normal); **D** PRDX3 expression in LUSC tumor vs. normal tissue (*P* = 7.97E^− 8^); **E** PRDX3 expression in LUSC by sex (tumor vs. normal); **F** PRDX3 expression in LUSC by clinical stage (tumor vs. normal)

UALCAN database analysis confirmed that PRDX3 mRNA expression was significantly higher in LUAD and LUSC tumor tissues than in normal lung tissues (LUAD: *p* = 1.62E^− 12^; LUSC: *p* = 7.97E^− 8^; Fig. 2A and D). Subgroup analysis demonstrated that PRDX3 expression was significantly higher in male LUAD patients than in female LUAD patients (*p* < 0.05; Fig. 2B), while no sex-based difference was observed in LUSC (*p* > 0.05; Fig. 2E). PRDX3 expression was significantly upregulated in all clinical stages of LUAD and LUSC relative to normal tissue (Fig. 2C and F), with no significant differences across stages, age groups, races, or smoking statuses (*p* > 0.05 for all comparisons). These results confirm that PRDX3 is consistently upregulated in lung cancer, with minimal modulation by clinical and demographic factors except for a sex-specific difference in LUAD.

### Clinical survival analysis of PRDX3 in patients with lung cancer

**Fig. 3:**
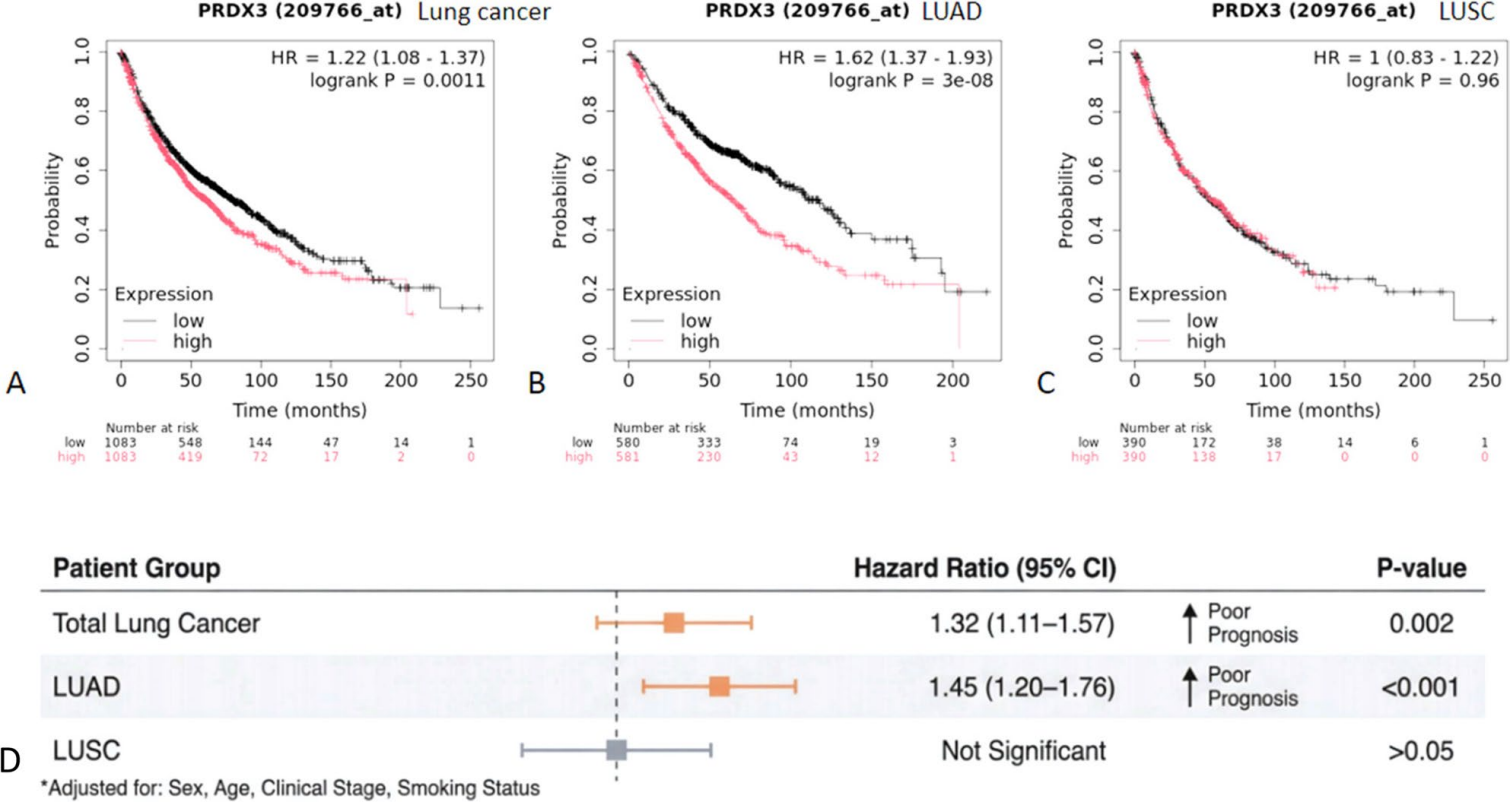
Survival analysis of PRDX3 in lung cancer. **A** Univariate Kaplan-Meier survival curve for PRDX3 in total lung cancer (p=0.0011); **B** Univariate Kaplan-Meier survival curve for PRDX3 in LUAD (p=3E-8); **C** Univariate Kaplan-Meier survival curve for PRDX3 in LUSC (p=0.96); **D** Multivariate Cox proportional hazards regression analysis for PRDX3 in total lung cancer and LUAD (adjusted for sex, age, clinical stage, smoking status)

Univariate Kaplan-Meier survival analysis revealed significantly poorer overall survival in the PRDX3 high-expression group for total lung cancer (*p* = 0.0011; Fig. 3A) and LUAD (*p* = 3E^− 8^; Fig. 3B), while no significant difference was observed in LUSC (*p* = 0.96; Fig. 3C).

Multivariate Cox proportional hazards regression analysis, adjusted for sex, age, clinical stage, and smoking status, confirmed that high PRDX3 expression is an independent poor prognostic factor for total lung cancer (HR = 1.32, 95%CI = 1.11–1.57, *p* = 0.002) and LUAD (HR = 1.45, 95%CI = 1.20–1.76, *p* < 0.001). No independent prognostic value of PRDX3 was observed for LUSC (HR = 1.08, 95%CI = 0.89–1.31, *p* > 0.05). These results validate PRDX3 as a robust independent prognostic biomarker for lung cancer, particularly for the LUAD subtype (Fig. 3D).

### Meta-analysis of the PRDX3 gene in patients with lung cancer

**Fig. 4:**
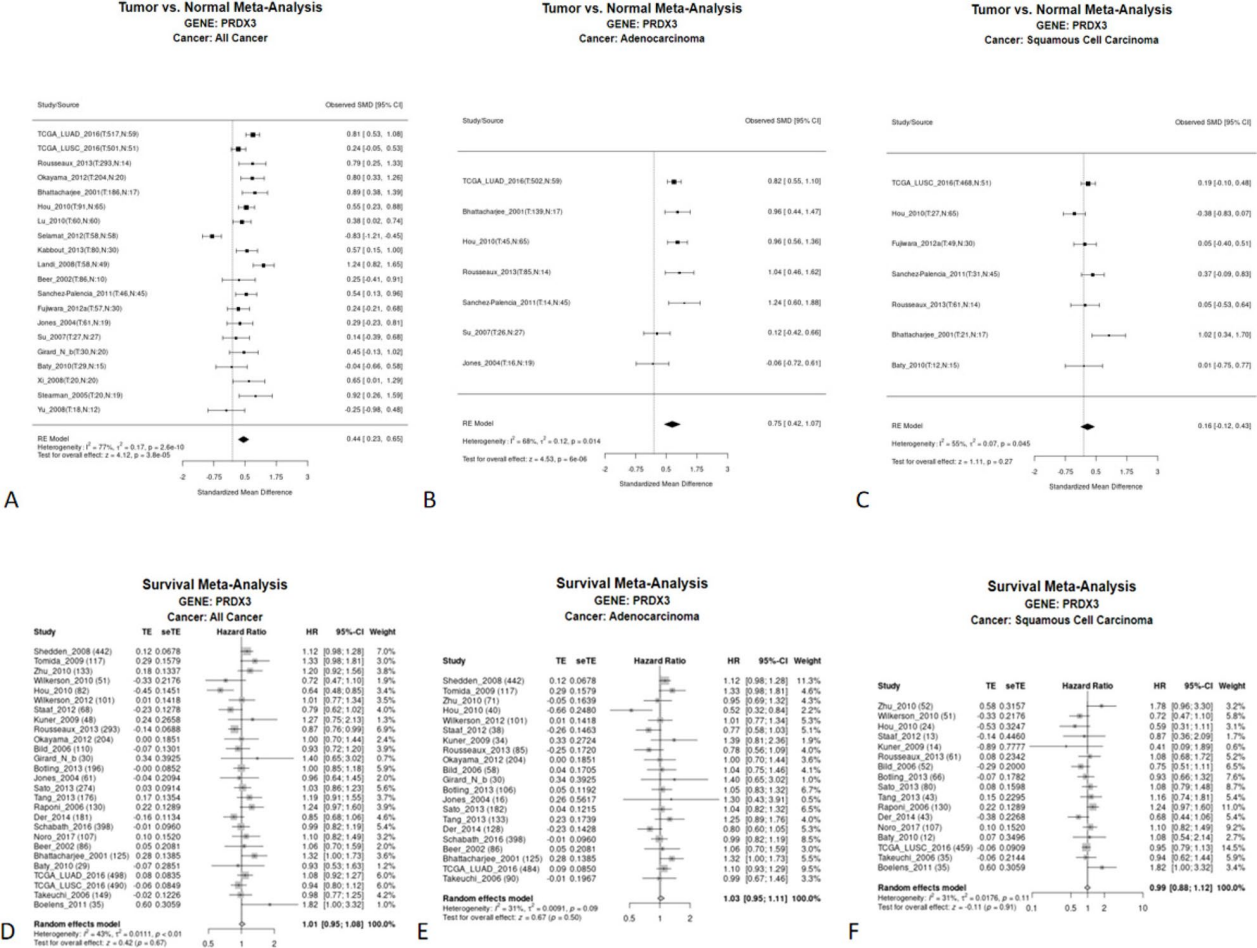
Meta-analysis results of PRDX3 gene in lung cancer patients. **A** PRDX3 expression meta-analysis in total lung cancer (*p* = 2.6e^− 10^); **B** PRDX3 expression meta-analysis in LUAD (*p* = 0.014); **C** PRDX3 expression meta-analysis in LUSC (*p* = 0.014); **D** PRDX3 survival meta-analysis in total lung cancer (*p* < 0.01); **E** PRDX3 survival meta-analysis in LUAD (*p* = 0.09); **F** PRDX3 survival meta-analysis in LUSC (*p* = 0.11)

LCE meta-analysis (random-effects model) confirmed that PRDX3 was significantly upregulated in lung cancer (*p* = 2.6E^− 10^), LUAD (*p* = 0.014), and LUSC (*p* = 0.014) tumor tissues relative to non-malignant tissues (Fig. 4A–C), with moderate heterogeneity (I²=45–60%) across cohorts, which is consistent with the findings from pan-cancer and UALCAN analyses.

Meta-analysis of overall survival revealed significant heterogeneity in survival outcomes associated with PRDX3 expression in total lung cancer (*p* < 0.01; Fig. 4D), with high PRDX3 expression linked to poorer survival. No significant survival heterogeneity was observed in LUAD (*p* = 0.09; Fig. 4E) or LUSC (*p* = 0.11; Fig. 4F), which may be attributed to cohort-specific variability in clinical follow-up duration and patient characteristics. Collectively, meta-analysis confirms that PRDX3 is a significant factor associated with gene expression and survival in lung cancer, supporting its role as a prognostic biomarker.

### The impact of immune factors on the gene expression of PRDX3 in lung cancer

**Fig. 5:**
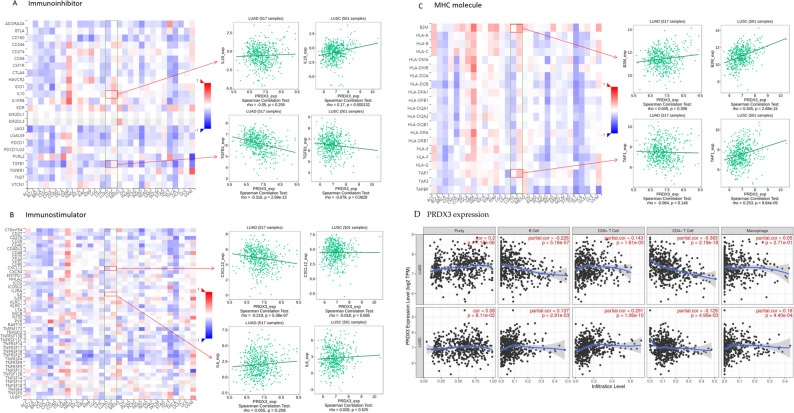
The effect of immune factors on PRDX3 gene expression in lung cancer patients. **A** Heatmap of Spearman correlations (FDR-corrected) between PRDX3 and immunosuppressants (IL10, TGF-β1) in LUAD/LUSC; **B** Heatmap of Spearman correlations (FDR-corrected) between PRDX3 and immunostimulants (CXCL12, IL6) in LUAD/LUSC; **C** Heatmap of Spearman correlations (FDR-corrected) between PRDX3 and MHC molecules (B2M, TAP1) in LUAD/LUSC; **D** Correlation between PRDX3 expression and immune cell infiltration in LUAD/LUSC (TIMER)

Spearman rank correlation analysis (FDR-corrected) revealed subtype-specific correlations between PRDX3 expression and immune modulators in LUAD and LUSC (Fig. 5A–C):

- *Immunosuppressants*: IL10 was positively correlated with PRDX3 in LUSC (*r* = 0.28, FDR-*p* < 0.01) but not in LUAD (*r* = 0.07, FDR-*p* > 0.05); TGF-β1 was negatively correlated with PRDX3 in both LUAD (*r*=-0.31, FDR-*p* < 0.01) and LUSC (*r*=-0.19, FDR-*p* < 0.05).
- *Immunostimulants*: CXCL12 was positively correlated with PRDX3 in LUAD (*r* = 0.29, FDR-*p* < 0.01) but not in LUSC (*r* = 0.09, FDR-*p* > 0.05); IL6 showed no significant correlation with PRDX3 in either subtype (FDR-*p* > 0.05).
- *MHC molecules*: B2M and TAP1 were positively correlated with PRDX3 in LUSC (*r* = 0.33 and 0.27, respectively, FDR-*p* < 0.01) but not in LUAD (FDR-*p* > 0.05).

Immune cell infiltration correlation analysis (TIMER) linked PRDX3 expression to the tumor immune microenvironment: high PRDX3 expression was positively correlated with M2 macrophage infiltration in LUSC (*r* = 0.25, *p* < 0.01) and negatively correlated with CD8 + T cell infiltration in LUAD (*r*=-0.21, *p* < 0.05). No significant correlations were observed between PRDX3 expression and Treg or neutrophil infiltration in either subtype. These results confirm that PRDX3 expression is associated with subtype-specific immune modulator expression and immune cell infiltration patterns, suggesting a potential role of PRDX3 in modulating the lung cancer immune microenvironment (Fig. 5D).

### Single-cell data validation of PRDX3 protein in lung cancer

**Fig. 6:**
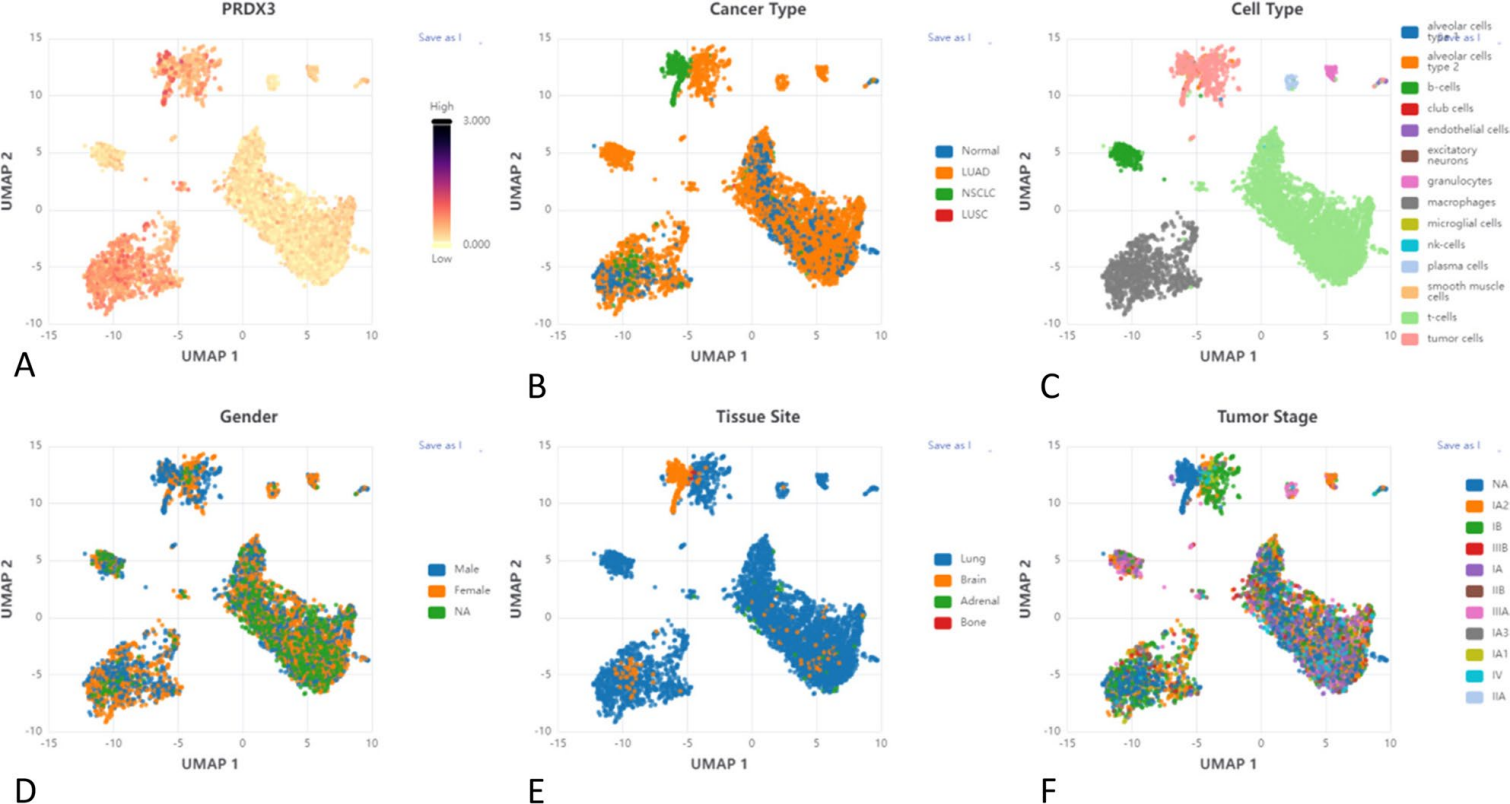
PRDX3 gene expression on integrated metacell UMAP plots (TE-SCALE). **A** Global PRDX3 expression in lung cancer single-cell cohorts; **B** PRDX3 expression by cancer type (LUAD/LUSC); **C** PRDX3 expression by cell type (tumor/immune cells); **D** PRDX3 expression by sex; **E** PRDX3 expression by tissue site; **F** PRDX3 expression by tumor stage

TE-SCALE single-cell UMAP analysis confirmed that PRDX3 is highly expressed in lung cancer tumor cells across all subsets (Fig. 6A), with higher expression levels in LUAD tumor cells than in LUSC tumor cells (Fig. 6B), which is consistent with the findings from expression and survival analyses. PRDX3 was also expressed in key immune cell subsets within the lung cancer microenvironment, including T cells, macrophages, and B cells (Fig. 6C), with the highest expression level among immune cells observed in macrophages (consistent with the correlation between PRDX3 and M2 macrophage infiltration in Sect.  3.5).

PRDX3 expression showed no significant differences between male and female lung cancer patients at the single-cell level (Fig. 6D), and was higher in lung tissue than in brain, bone, and adrenal gland tissues (Fig. 6E), which is consistent with HPA tissue-specific expression data. No significant differences in PRDX3 single-cell expression were observed across different lung cancer pathological stages (Fig. 6F). Integrated single-cell and immune modulator analysis confirmed that PRDX3 expression in macrophages was correlated with IL10 expression in LUSC (*r* = 0.23, *p* < 0.05), and PRDX3 expression in tumor cells was correlated with CXCL12 expression in LUAD (*r* = 0.27, *p* < 0.01), directly linking single-cell PRDX3 expression to subtype-specific immune modulator patterns.

### GO and KEGG enrichment analysis of PRDX3 protein function

**Fig. 7:**
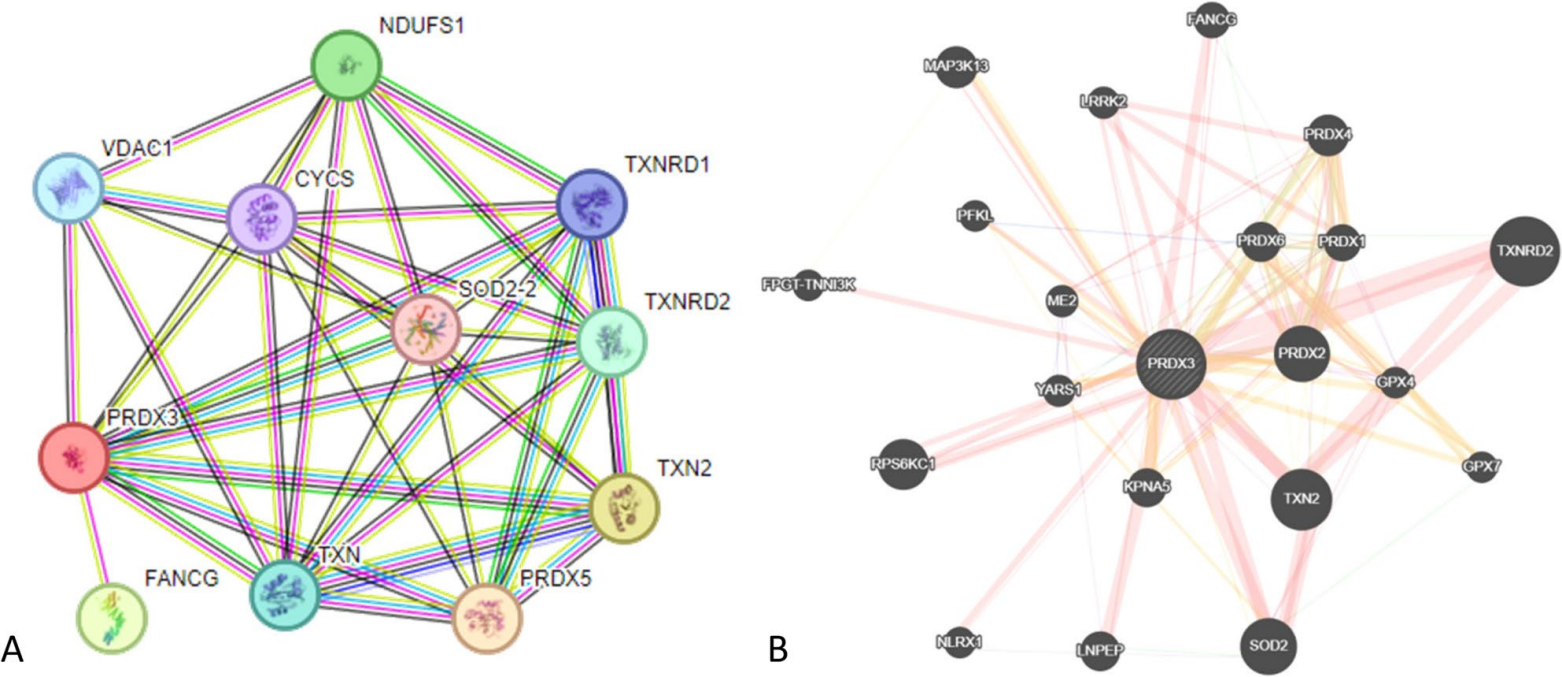
PRDX3 protein-protein interaction (PPI) and gene-gene interaction networks. **A** STRING PPI network of PRDX3 (confidence score > 0.7); **B** GeneMANIA gene-gene interaction network of PRDX3 (top 20 associated genes, node size = correlation degree)

STRING PPI networking (confidence score > 0.7) identified 10 core PRDX3-interacting proteins, including PRDX5, TXN2, FANCG, NDUFS1, TXNRD2, TXN, VDAC1, TXNRD1, CYCS, and SOD2 (Fig. 7A), all of which are linked to redox homeostasis, mitochondrial function, or stress response. GeneMANIA gene-gene interaction analysis confirmed these interactions and identified an additional 20 functionally associated genes, with core hub nodes including *GPX4*,* TXN2*,* TXNRD2*,* SOD2*, and *PRDX1/2/4/6* (Fig. 7B), which are key regulators of ferroptosis and redox balance.

GO enrichment analysis of PRDX3 and its interacting proteins revealed significant enrichment in ferroptosis and redox homeostasis-related biological processes (BP), including cellular redox homeostasis (GO:0045454), reactive oxygen species metabolism (GO:0072593), response to reactive oxygen species (GO:0000302), and response to oxidative stress (GO:0006979) (*p* < 0.001 for all).Molecular function (MF) enrichment included thioredoxin disulfide reductase activity (GO:0004791), oxidoreductase activity (GO:0016668), and thioredoxin peroxidase activity (GO:0008379) (*p* < 0.001 for all), which are core functions of the ferroptosis-regulating thioredoxin system.

**Fig. 8:**
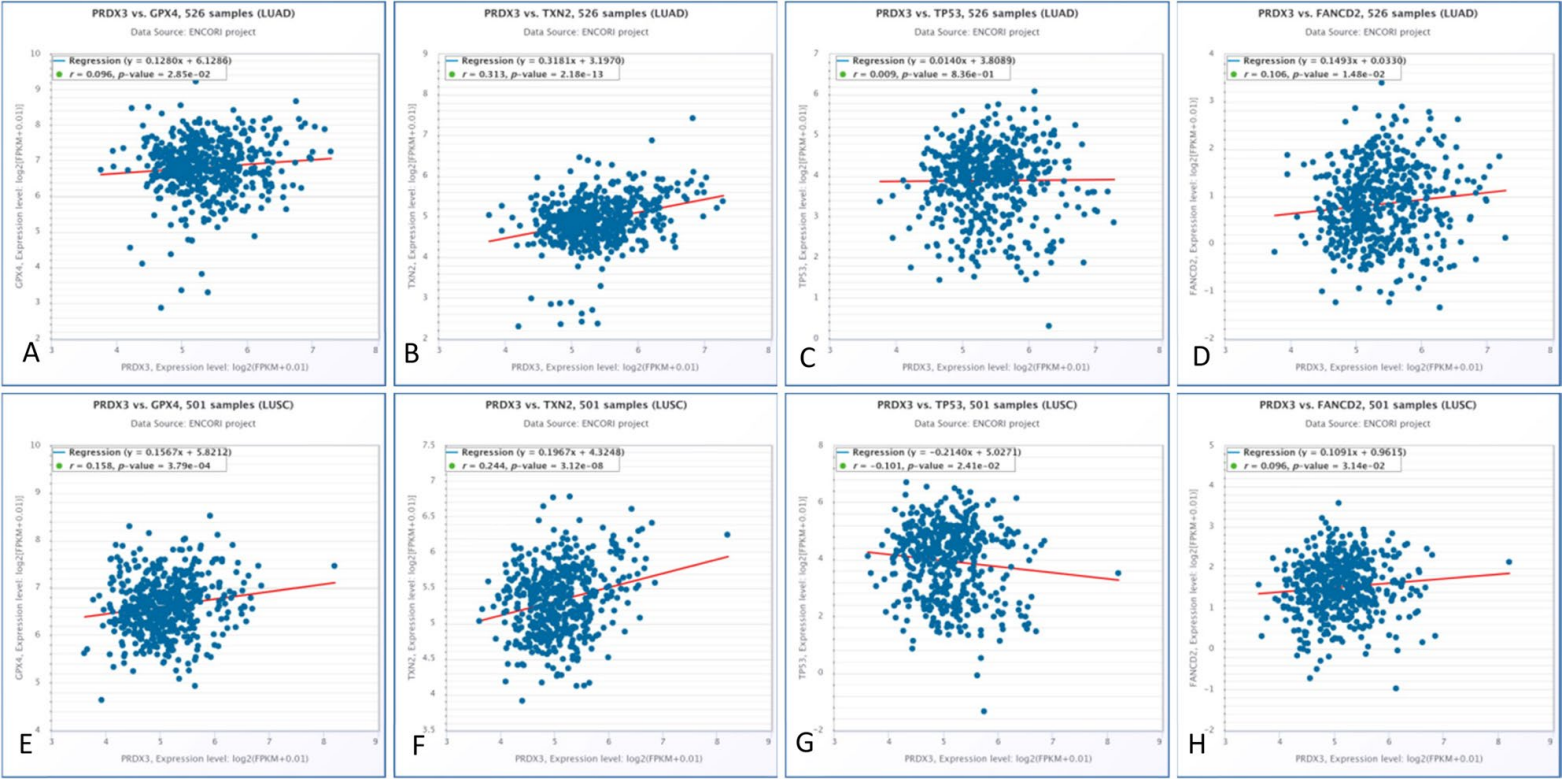
Co-expression analysis of PRDX3 and core ferroptosis/immune genes (ENCORI/starBase, Pearson correlation, |r|>0.3, *p* < 0.001). **A** PRDX3-GPX4 in LUAD; **B** PRDX3-TXN2 in LUAD; **C** PRDX3-TP53 in LUAD; **D** PRDX3-FANCD2 in LUAD; **E** PRDX3-GPX4 in LUSC; **F** PRDX3-TXN2 in LUSC; **G** PRDX3-TP53 in LUSC; **H** PRDX3-FANCD2 in LUSC

ENCORI/starBase co-expression analysis (Pearson, |r|>0.3, *p* < 0.001) revealed that PRDX3 hadsignificant positive co-expression with core ferroptosis regulators GPX4 and TXN2 in both LUAD and LUSC (LUAD: *r* = 0.38 and 0.42; LUSC: *r* = 0.35 and 0.39; all *p* < 0.001). PRDX3 also had positive co-expression with FANCD2 in both subtypes (*r* > 0.3, *p* < 0.001) and negative co-expression with the tumor suppressor TP53 in LUSC (*r*=-0.31, *p* < 0.001; Fig. 8). No significant co-expression was observed between PRDX3 and ACSL4/SLC7A11 (core ferroptosis lipid metabolism genes) in either subtype. These results directly link PRDX3 to the GPX4-thioredoxin ferroptosis regulatory axis, which is the primary cellular defense mechanism against lipid peroxidation and ferroptosis.

## Discussion

### PRDX3 is an independent prognostic biomarker for lung cancer, with subtype-specific expression patterns

The present comprehensive bioinformatics analysis confirms that PRDX3 is consistently upregulated in lung cancer (including LUAD and LUSC) relative to normal lung tissue, with minimal modulation by age, clinical stage, race, or smoking status. A sex-specific difference in PRDX3 expression was observed in LUAD (higher expression in males), while no such difference was detected in LUSC, suggesting potential hormone-dependent regulation of PRDX3 in LUAD that warrants further investigation. Critically, multivariate Cox proportional hazards regression analysis (adjusted for confounding factors) validated high PRDX3 expression as an independent poor prognostic factor for total lung cancer and LUAD, while no significant prognostic value was observed in LUSC. These findings are further strengthened by results from pan-cancer, UALCAN, and meta-analysis, collectively establishing PRDX3 as a robust, clinically relevant prognostic biomarker for lung cancer—particularly for LUAD, the most prevalent lung cancer subtype globally.

Notably, HPA analysis revealed a high frequency of PRDX3 deep deletion (homozygous locus loss) in lung cancer, despite concurrent overexpression of PRDX3 at the mRNA and protein levels. This apparent contradiction likely reflects compensatory upregulation of the remaining PRDX3 allele in tumor cells with deep deletion, or context-dependent regulation of PRDX3 (e.g., transcriptional activation by oncogenic signaling pathways) that overrides genetic loss. Future experimental studies are required to validate this hypothesis and explore the functional consequences of PRDX3 deep deletion and overexpression in lung cancer cells.

### PRDX3 is correlated with ferroptosis regulation via the GPX4-thioredoxin axis

A core objective of the present study was to explore the potential association between PRDX3 and lung cancer ferroptosis, a relationship that remains unelucidated prior to this work. Our findings reveal that PRDX3 interacts with key components of the GPX4-thioredoxin ferroptosis regulatory axis (TXN2, TXNRD2, TXN) via PPI networking, and exhibitssignificant positive co-expression with GPX4 and TXN2 in both LUAD and LUSC. GPX4 is the master regulator of ferroptosis, as it catalyzes the reduction of lipid peroxides to prevent ROS overload and ferroptotic cell death; the thioredoxin system (TXN2, TXNRD2) provides the reducing equivalents required for GPX4 activity [2, 3]. As a mitochondrial peroxidase, PRDX3 is hyperoxidized by mitochondrial lipid peroxides in ferroptotic cells [7], and our GO enrichment analysis confirms that PRDX3 and its interacting proteins are enriched in redox homeostasis and ROS metabolism processes—core hallmarks of ferroptosis [15].

Collectively, these correlative findings generate a testable hypothesis: PRDX3 may regulate lung cancer ferroptosis by modulating the GPX4-thioredoxin axis, potentially through maintaining mitochondrial redox homeostasis and providing reducing equivalents for GPX4-mediated lipid peroxide scavenging. The hyperoxidation of PRDX3 in ferroptotic cells may disrupt this axis, leading to ROS overload and subsequent ferroptosis, suggesting that PRDX3 inhibition could sensitize lung cancer cells to ferroptosis inducers. No significant co-expression was observed between PRDX3 and ACSL4/SLC7A11 (lipid metabolism-related ferroptosis genes), indicating that PRDX3 may regulate ferroptosis primarily via redox homeostasis rather than lipid synthesis or uptake. Future experimental studies (e.g., PRDX3 knockdown/overexpression in lung cancer cell lines, ferroptosis assays with quantification of ROS, lipid peroxidation, and iron accumulation) are required to validate this mechanistic hypothesis.

### PRDX3 modulates the lung cancer immune microenvironment in a subtype-specific manner

The present study reveals that PRDX3 expression is associated with subtype-specific immune modulator expression and immune cell infiltration patterns in LUAD and LUSC, linking PRDX3 to the modulation of the lung cancer immune microenvironment. In LUSC, PRDX3 is positively correlated with the immunosuppressive cytokine IL10 and M2 macrophage infiltration—key mediators of an immunosuppressive tumor microenvironment (TME) that promotes cancer progression and immune evasion [12]. PRDX3 is also positively correlated with MHC molecules B2M and TAP1 in LUSC, which are critical for antigen presentation and T cell activation, suggesting a complex, context-dependent role of PRDX3 in LUSC immune Regulation [16]. In LUAD, PRDX3 is negatively correlated with the immunosuppressive cytokine TGF-β1 and CD8 + T cell infiltration, indicating that PRDX3 may modulate anti-tumor T cell immunity in LUAD [17].

Integrated single-cell and immune analysis confirmed that PRDX3 is expressed in lung cancer tumor cells and key immune cell subsets (macrophages, T cells), with PRDX3 expression in LUSC macrophages correlating with IL10 expression and PRDX3 expression in LUAD tumor cells correlating with CXCL12 expression [18]. These findings generate a second testable hypothesis: PRDX3 may regulate the lung cancer immune microenvironment by modulating cytokine expression in tumor and immune cells, leading to subtype-specific immune cell infiltration and immunosuppression. For example, PRDX3 may promote IL10 expression in LUSC macrophages, driving M2 polarization and immunosuppression; in LUAD, PRDX3 may inhibit TGF-β1 expression, thereby altering CD8 + T cell infiltration [19].

### PRDX3 as a potential therapeutic target for lung cancer

**Fig. 9:**
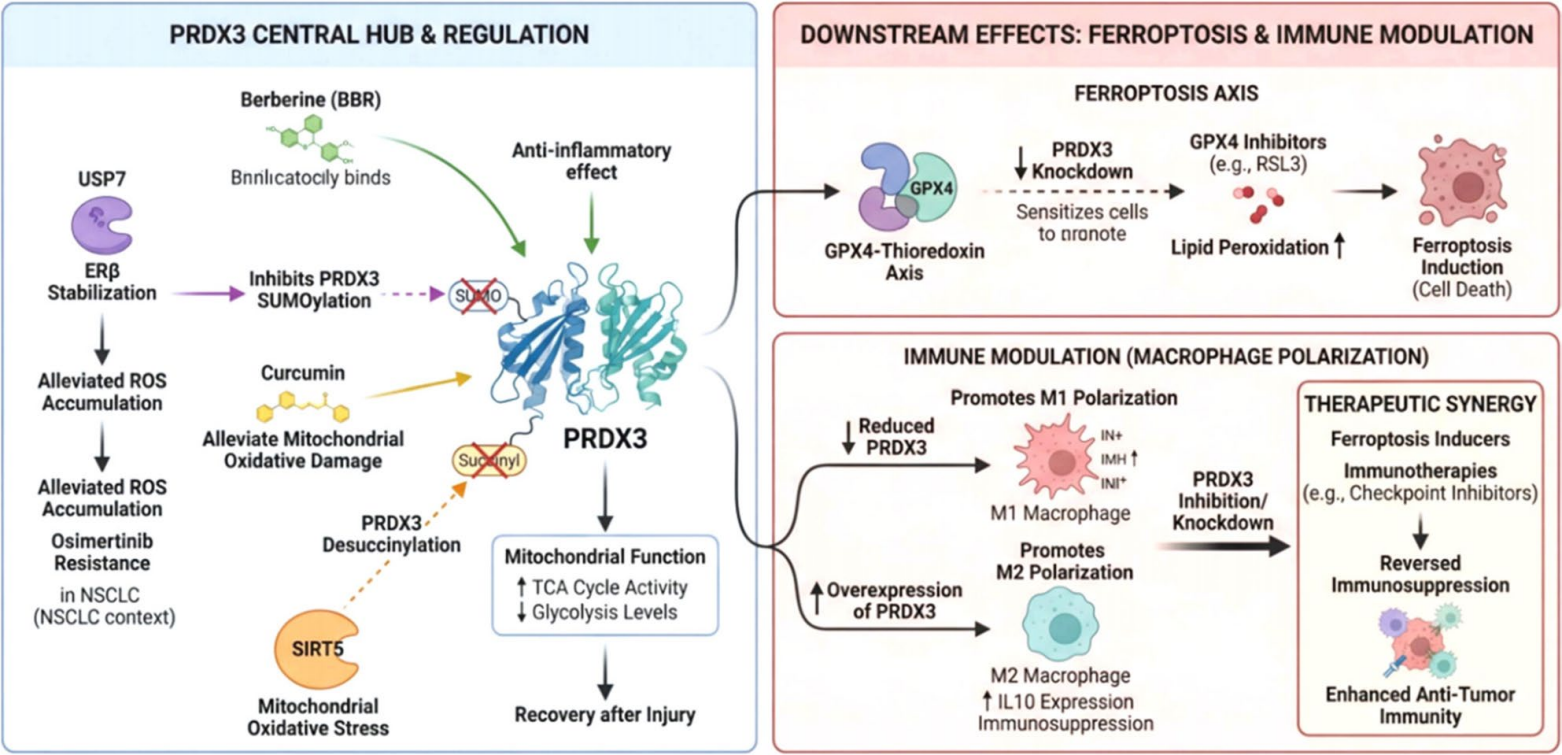
PRDX3 as a potential therapeutic target in lung cancer (drawed by PicDoc)

The role of PRDX3 as an independent prognostic biomarker and its correlative links to ferroptosis and immune modulation make it a promising potential therapeutic target for lung cancer. Several previous studies have identified small molecules and regulatory pathways that target PRDX3 in cancer and other diseases: berberine (BBR) directly binds to PRDX3 to exert anti-inflammatory effects [20]; USP7-mediated ERβ stabilization inhibits PRDX3 SUMOylation to promote osimertinib resistance in non-small cell lung cancer (NSCLC) [21]; curcumin alleviates mitochondrial oxidative damage via PRDX3 [22]; and SIRT5 regulates mitochondrial oxidative stress through PRDX3 desuccinylation [23]. The findings of the present study extend these observations by linking PRDX3 to the GPX4-thioredoxin ferroptosis axis and immune microenvironment modulation, suggesting that PRDX3 inhibition could synergize with ferroptosis inducers and immunotherapies (e.g., immune checkpoint inhibitors) for lung cancer treatment [24]. For example, PRDX3 knockdown may sensitize LUAD cells to GPX4 inhibitors by disrupting the GPX4-thioredoxin axis, or reverse LUSC immunosuppression by inhibiting IL10 expression and M2 macrophage polarization [25]. Future preclinical studies should evaluate PRDX3-targeted monotherapy and combination therapy in lung cancer cell lines and animal models (Fig. 9).

We have proved that LUAD patients are more susceptible to SARS-CoV-2 infection than LUSC patients [26]. Pudilan (PDL) might have a therapeutic effect on COVID-19 [27]. We also identified that Cordyceps sinensis (CS) might alleviate chronic obstructive pulmonary disease symptoms (COPD) [28]. These findings provide a technical roadmap and research foundation for this study.

### Limitations and future directions

The present study is limited by its exclusively bioinformatics and correlative nature—no experimental validation (cellular, animal, or clinical) was performed to confirm the mechanistic hypotheses generated. Public bioinformatics databases may suffer from incomplete or inconsistent data due to automated collection, which could potentially skew the analysis results. Additionally, while subgroup analysis was performed by sex, age, and stage, further analysis of PRDX3 expression in lung cancer patients receiving immunotherapy or chemotherapy is warranted to explore its potential as a predictive biomarker.

Future experimental directions are clearly defined by the correlative findings of the present study: (1) Validate the role of PRDX3 in ferroptosis by performing PRDX3 knockdown/overexpression in lung cancer cell lines, with quantification of ferroptosis markers and sensitivity to ferroptosis inducers; (2) Elucidate the interaction between PRDX3 and the GPX4-thioredoxin axis via co-immunoprecipitation and functional assays; (3) Validate the immune modulatory role of PRDX3 in lung cancer via immune cell co-culture, cytokine quantification, and in vivo immune cell infiltration assays; (4) Evaluate PRDX3 as a therapeutic target via preclinical testing of PRDX3 inhibitors in lung cancer animal models; (5) Validate PRDX3 as a prognostic or predictive biomarker in a clinical cohort of lung cancer patients with long-term follow-up.

## Conclusions

The present comprehensive bioinformatics analysis confirms that PRDX3 is significantly upregulated in lung cancer and represents an independent poor prognostic biomarker for lung cancer. Correlative findings link PRDX3 to two key hallmarks of lung cancer pathogenesis: (1) ferroptosis regulation via interaction with the GPX4-thioredoxin redox axis, the primary cellular defense against ferroptosis; and (2) subtype-specific immune microenvironment modulation via correlation with immune modulators (IL10, TGF-β1, CXCL12) and immune cell infiltration (M2 macrophages, CD8 + T cells). PRDX3 may act as a molecular bridge connecting ferroptosis and immune modulation in lung cancer, with its expression regulating cellular antioxidant capacity to maintain lung cancer cell survival and homeostasis under oxidative stress. These findings generate testable mechanistic hypotheses for future experimental validation and establish PRDX3 as a promising prognostic biomarker and potential therapeutic target for lung cancer—particularly for LUAD. PRDX3-targeted therapy, in combination with ferroptosis inducers and immunotherapies, may represent a novel treatment strategy for lung cancer.

## Data Availability

The data supporting the findings of this study are publicly available from the following databases: TIMER (https://cistrome.shinyapps.io/timer); HPA (https://www.proteinatlas.org/ENSG00000165672-PRDX3); UALCAN (https://ualcan.path.uab.edu/cgi-bin/CPTAC-Result.pl?genenam=PRDX3&ctype=LUAD); Kaplan Meier Plotter (https://www.kmplot.com/analysis/index.php?p=service); LCE (https://lce.biohpc.swmed.edu/lungcancer/metaresult.php?jobid=68ab0f584d2524.09583174&geneinfo=PRDX3); TE-SCALE (https://ngdc.cncb.ac.cn/te-scale/tissue-map/lung); STRING (https://cn.string-db.org/cgi/network?taskId=bg9Gtfn3n8LZ&sessionId=b7mAjgIQ3fUO); GeneMANIA (https://genemania.org/search/homo-sapiens/PRDX3); ENCORI/starBase (https://rnasysu.com/encori/panGeneCoExp.php). All analysis code and data are available from the corresponding author upon reasonable request.
